# Fatal attraction: rare species in the spotlight

**DOI:** 10.1098/rspb.2008.1475

**Published:** 2009-01-13

**Authors:** Elena Angulo, Anne-Laure Deves, Michel Saint Jalmes, Franck Courchamp

**Affiliations:** 1 Ecologie, Systématique et Evolution, UMR CNRS 8079, Univ Paris Sud91405 Orsay Cedex, France; 2 Muséum National d'Histoire Naturelle, UMR 5173 MNHN-CNRS-Paris VI, Département des Jardins Botaniques et Zoologiques, Ménagerie du Jardin des Plantes57 rue Cuvier, 75005 Paris, France

**Keywords:** anthropogenic Allee effect, conservation, rarity, species value, willingness to pay, zoological garden

## Abstract

The exploitation of rare and endangered species can end in the species's extinction because the increased value people associate with rarity increases the economic incentive to exploit the last individuals, creating a positive feedback loop. This recently proposed concept, called the anthropogenic Allee effect (AAE), relies on the assumption that people do value rarity, but this remains to be established. Moreover, it also remains to be determined whether attraction to rarity is a trait confined to a minority of hobbyists (e.g. wildlife collectors, exotic pet owners) or characteristic of the general public. We estimated how much the general public valued rare species compared with common ones, using five different metrics related to personal investment: time spent, physical effort, unpleasantness, economic investment and risk. We surveyed the visitors of a zoo. To see the rare species, the visitors to the zoo invested more time in searching and contemplation, they were ready to expend more physical effort, they tolerated more unpleasant conditions, they were willing to pay more and, finally, they risked more to obtain (steal) a rare species. Our results provide substantial evidence of how the general public places more value on rare species, compared with common species. This confirms the AAE as an actual process, which in addition concerns a large part of the population. This has important consequences for the conservation of species that are rare now, or that could become so in the future.

## 1. Introduction

Among the chief factors responsible for the current catastrophic decline of biodiversity is overexploitation in all its forms (Rosser & Mainka 2002). Many species are known to be overexploited, but, until recently, logic and economic theory suggested that rare species would be safe from this threat, as the high cost of exploiting rare species would render their continued exploitation as non-economically viable (Clark 1990). This theory has, however, recently been challenged by a new concept, named the anthropogenic Allee effect (AAE). According to this concept, the abstract value people attribute to rarity would confer an economical value to the rare species that would maintain the incentive to exploit them, even at very high levels of rarity (Courchamp *et al*. 2006). Rare species being more valuable, they would be more exploited, and thereby become even rarer and even more valuable, precipitating them into a vortex of extinction.

In their study on this new concept, Courchamp *et al*. (2006) presented the theory with a mathematical model of environmental economy, and several factual data supported the examples concerning different types of human activity (such as collections, trophy hunting, traditional medicine, exotic pets, luxury goods or even ecotourism). Although the demonstration was realistic, the keystone of the reasoning was still missing: the whole theory relies on the assumption that people do value rarity, but this remains to be established. One of the problems when describing the AAE concept was to distinguish between correlation and causation when searching for examples of the relationship between rarity and value (Courchamp *et al*. 2006). One possibility of solving this problem and actually testing the effect could be tracking changes in a particular species's demand curve with rarity. For example, it has been shown that fleet size engaged in whale watching increased as killer whales' (*Orcinus orca*) abundance decreased (Bain 2002); also, caviar price in markets increased as sturgeon abundance decreased (Gault *et al*. 2008). However, these types of tests are specific and lack generalization to other situations. In fact, another important problem when attempting to demonstrate the relationships between price and rarity value (and thus demonstrate an AAE) is cross-species comparison (Slone *et al*. 1997; Jepson & Ladle 2005). Jepson & Ladle (2005) showed how the value of some bird species is sufficient to interest investors willing to overcome the technical challenges that commercial breeding of these species may pose; different motivations and preferences of bird-keeping hobbyists would change relationships between price and rarity for different species. To test truly the AAE, the species of attention (which gains value as it becomes rarer) should be comparable between common and rare species—especially reducing bias owing to colour, form or size. It also remains to be determined whether attraction of rarity is a trait confined to a minority of hobbyists or characteristic of the general public. In the latter case, the implication would be a potentially much wider impact of the AAE.

Here, we performed a set of rigorous experiments to estimate how many people valued rare species compared with common ones—we hypothesized that people will value rare species more than common ones. We assumed that the value was proportional to personal investment, and estimated such investment with different metrics. We tested the visitors of a zoo to assess the value they put on rarity in animal species, using a gradient of investment: time spent, physical effort, unpleasantness, economic investment and willingness to steal.

## 2. Material and methods

### (a) Study site

We performed the experiments in the zoological garden La Ménagerie du Jardin des Plantes, Paris (France). This is the oldest zoo in France (opened in December 1794) and is linked to the scientific research institute, Le Muséum National d'Histoire Naturelle.

### (b) Time investment in observing a rare species

We performed this experiment in the vivarium, where we placed two different terrariums behind a window. They were placed sufficiently far apart that people could look either at one or at the other, but not both simultaneously. In each one, one could see at any given time between 15 and 20 individuals of a dendrobatid frog species. A panel alongside each terrarium informed visitors of the rarity of the species: one indicated the presence of a common species, and the other a rare species. Terrariums were large and frogs could be easily located, so we measured the time people invested observing the individuals. The animals used were two subspecies of *Dendrobates tinctorius* that are very similar; pictures of each subspecies were added in both panels.

We regularly interchanged the two information panels (which indicated that the species was either rare or common) between both terrariums, to remove a possible effect of the terrarium. We recorded with a chronometer the time each visitor spent observing each of the terrariums. The terrariums were rather low, so that people had to bend to observe the frogs. Time began to run for each person when they adopted this position. We only recorded visitors who were alone, who observed both terrariums and who had read the information panels before viewing the exhibition. We only recorded the time for the visitors who entered the room when no other visitor was observing either terrarium. We performed this experiment during three weeks in August 2006. Because in this case the direction of the visit could influence the results, we also recorded for each visitor the direction of their visit (whether the first terrarium they looked at was the left one or the right one) and the position of the information panels (whether the rare species was on the left or on the right terrarium).

To statistically analyse the data, we first tested all the potential candidate distribution of errors for the dependent variable, to select the one that minimized the deviance of the model (Herrera 2000). In this case, the dependent variable was the time observing the animal; we tested the Poisson, gamma and negative binomial distributions and we finally used generalized linear models with negative binomial distributions and log link (GLM_NB_). The main independent variable was the type of species observed (common or rare). Because we wanted to compare differences in time within each observer, the observer was introduced as a repeated measure in GLM_NB_. We performed separate tests for each visit direction (whether the visitor looked first at the right or left terrarium) and for each information panel location (whether the rare species was in the right or left terrarium). Here, we hypothesized that the more valuable the animal was to the visitor, the longer they would spend taking pleasure (or interest) in watching it.

### (c) Time investment in searching for a rare species

We performed this experiment in the reptile gallery. We used the same protocol described previously, with two different and independently spaced terrariums with panels above each terrarium. This time terrariums were opened to the public by a small window of 30×20 cm, but were long (1 m) and heavily decorated with plants, small branches and rocks. We added pictures of two different animals of the same species in each panel and indicated that one was rare and the other common (as above). We performed this experiment with a panel indicating the presence of either a dendrobatid frog (two weeks) or a *Phelsuma* gecko (two weeks); however, no animals were inside. Even if both the terrariums were very similar in decoration, we regularly interchanged the ‘rare’ and ‘common’ information panels between both terrariums every 2 days, to remove a possible effect of the terrarium. We hypothesized that the more valuable the animal, the longer people would spend looking for it.

During four weeks in March and April 2006, we recorded the time each visitor remained looking at each of the terrariums in the same conditions as the previous experiment. In addition, we recorded for each visitor whether the first terrarium they observed was the one indicating the presence of the rare or common species. As in the previous experiment, we analysed the data with a GLM_NB_ in which the dependent variable was the time searching for the animal; the visitor was introduced as a repeated measure and the main independent variable was the type of species (common or rare). We added one more independent variable: the first terrarium observed (whether the visitor looked first at the rare or common terrarium) and its interaction with the main independent variable. We performed different tests for both of the species (frog and gecko).

### (d) Physical effort investment to see a rare species

In one of the display rooms (the big-cat house) of La Ménagerie Zoo, we put a panel on a closed door to inform visitors that either a common or rare species could be seen behind the door. Depending on the day, the panel indicated that the display of this species was on either the ground, first, second or third floor. To attract people to the panel, a photo of the eastern chipmunk (*Tamias striatus*) was clearly visible. We chose it as it is a relatively well-known species in western Europe, interesting to observe and could pass for either a rare or common species. During four weeks, we measured how many people chose to try to open the door or left without trying to open the door after having read the panel. Each day, we changed the panel to obtain observations for at least 2 days with each combination of rare/common species and the four floors. We did not consider groups with children or people likely to have difficulties climbing the stairs (old or disabled visitors).

We used a generalized linear model with a binomial distribution and logit link function (GLM_B_) corresponding to the distribution of the dependent variable: the number of visitors or groups of visitors trying to open the door per day with respect to the total number of people who read the panel. The independent variables were the type of species to see (rare or common) and the effort level (measured by the floor on which the species could be seen).

### (e) Tolerance of unpleasantness to see a rare species

To do this experiment, we chose an intersection between two paths in the far end of the zoo. In this intersection, we placed panels indicating in one direction the way to see either a ‘common species’ or a ‘rare species’, and in the other the ‘rest of the visit’. In the former path, we installed a water sprinkler so that the visitors who chose to take this path would get wet to arrive at the exhibit. For comparison, we also recorded data without sprinkling the path. We recorded the number of visitors who, having read the panel, chose either the common or rare direction (instead of the the ‘rest of the visit’), in the presence and absence of the sprinkler. The experiment was conducted over three weeks during July 2006, and we performed seven sessions of approximately 1–3 hours for each combination of species (common or rare) and unpleasantness (sprinkler switched on or off). We performed the experiment only in the morning to avoid the hot hours during the day (when people could welcome being showered). The intersection was chosen as a location in the zoo with few visitors, and we recorded only the visitors who were not following other visitors, so that they would not be influenced by previous visitor's decisions. We performed a GLM_B_ in which the dependent variable was the number of visitors or groups of visitors who took the path to see the species with respect to the total number of people who read the panels in each observation session. The independent variables were the type of species to see (rare or common) and the unpleasantness (measured by the water sprinkler switched on or off).

### (f) Economic investment to see a rare species

In this next experiment, we wanted to see how many visitors were willing to pay to see a rare species compared with a common species. We performed this experiment in the nursery building of the zoo. At the entrance door of the nursery, we installed a panel informing visitors that a common, or rare, species could be seen inside by paying an extra amount of money. We established four fees of €1, 2, 4 and 8. For reference, the entrance to the whole zoo already costs each visitor €7 (€5 for children). Each day, we changed the panel so that in 8 days we performed all combinations of two types of species (rare and common) and four extra fees. We started by the cheapest and ended with the most expensive fee with the same amount of display time for each category (one full day). We performed this experiment during four weekends in June 2007. We recorded the number of visitors or groups of visitors who, having read the panel, decided either to enter into the nursery or to continue the visit to other buildings. The entrance door was closed, so people entering had already decided to pay (many of them had the money already prepared). Thus, the fees were actual, but not cashed: once inside the nursery, we informed the visitors that this day the entrance was free because the species were unavailable to see. Other species were available for display in the nursery. We performed GLM_B_ in which the dependent variable was the number of visitors or groups of visitors who decided to enter into the nursery with respect to the total number of people who read the panel each day, and the independent variables were the type of species to see (rare or common) and the fee to pay (€1, 2, 4 or 8).

### (g) Risk exposure to get a rare species

In a final experiment, we wanted to test how many visitors would take a risk to obtain a rare species. We set up a display in the big cat house, behind a fence, in full public visibility and almost out of reach: a table with two jars of 500 seeds of *Vicia faba*. We selected an Indian variety that presents an unfamiliar shape and colour. We added a panel with two photos of the grown plant and a short explanation that the apparently identical seeds corresponded to two apparently identical plants that differ only in their rarity. Each jar was marked as rare/common species. We marked the seeds in the common jar to ascertain whether people had changed the seeds between the jars. We reversed the jar positions on the table halfway through the experiment, in order to avoid any influence of the jar position. The experiment took four weeks during July and August 2006. Each day, we counted the number of seeds that were left in each jar, to see how many seeds of each type were stolen. As for the two first experiments, we used a GLM_NB_, and in this case the dependent variable was the number of seeds stolen and the independent variables were the type of seed species (rare or common) and the jar position (right or left). We added date as a repeated measure, allowing a comparison of the stolen seeds of the common and rare species within each day.

We performed computations with Statistica v. 6.0 (StatSoft 2001) and the SAS package (Genmod v. 9.1.3; SAS Institute 2004).

## 3. Results

The amounts of effort (measured in different metrics, as explained above) the visitors were willing to exert for the rare and common species are reported in table 1, for all six experiments. The corresponding main statistics-associated significance levels are reported in table 2.

### (a) Time investment in observing a rare species

**Figure 1:**
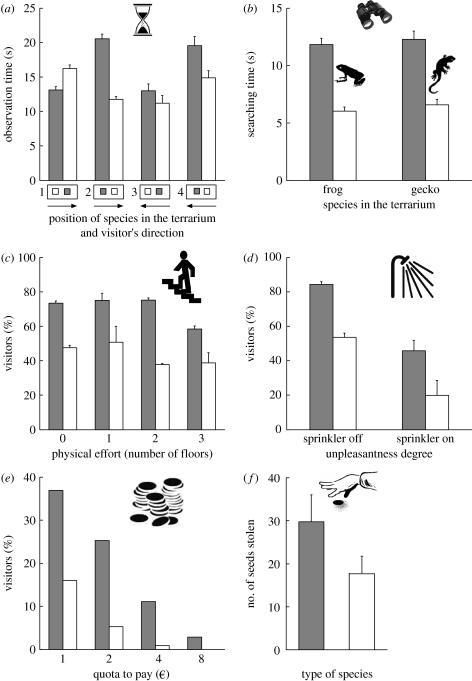
Effects of rare or common species on personal investment measures: the time dedicated by zoo visitors to (*a*) observe dendrobathid frogs and (*b*) search for non-existent frogs and geckos in a terrarium; the percentage of visitors who decided (*c*) to climb 0–3 floors to see the species, (*d*) to tolerate being sprinkled to see the species and (*e*) to pay an additional €1–8 to see the species; and (*f*) the number of seeds surreptitiously stolen by visitors. Grey bars, rare species; white bars, common species.

In general, 608 visitors spent more time observing what they thought was a rare species (table 1; figure 1*a*). However, the display was located at the end of the terrarium building, and the results were influenced by the visit direction: time invested contemplating the rare species was always higher, except in the case when visitors both made the visit backwards and started by the common species (table 2; figure 1*a*).

### (b) Time investment in searching for a rare species

The time searching for the non-existent animals was higher for the terrarium supposedly having a rare species (tables 1 and 2; figure 1*b*). Whether the first terrarium observed was the one with the rare or common species did not significantly influence the time spent searching (*p*>0.5 in both cases).

### (c) Effort investment to see a rare species

In total, 856 visitors or groups of visitors read the panel that indicated the level of physical effort (climb the stairway or not) and the reward (level of rarity of the species). We recorded an average of 100 persons per combination of rare/common species and floors. The percentage of visitors trying to open the door was significantly related to the type of species (table 2) and the effort level (*F*_3,15_=23.30, *p*<0.001). As the effort increased (higher floors), fewer visitors were willing to climb the stairs (table 1). Yet, regardless of the effort level, more of them tried for the rare species (figure 1*c*).

### (d) Tolerance of unpleasantness to see a rare species

The visitors (1369) or groups of visitors arriving at the intersection evaluated both the type of species to see and the unpleasantness of getting wet when the sprinkler was on. We obtained an average of 340 persons per combination of rare/common species and water sprinkler switched on or off. The percentage of visitors taking the path to see the species was significantly related to the type of species (table 2) and unpleasantness (*F*_1,23_=53.25, *p*<0.001). More visitors took the path to see the species when the information panel indicated that a rare species could be seen, even when the unpleasantness of the water sprinkler was incorporated (table 1; figure 1*d*). When the water sprinkler was switched on, a lower number of visitors took this path than when it was off, but these numbers were proportionally smaller for the rare species (36%) than for the common species (46%): more visitors were discouraged by the sprinkler when the species was common than when it was rare. Thus, visitors were more likely to tolerate being sprinkled to see a rare species than for a common species.

### (e) Economic investment to see a rare species

In total, 895 visitors or groups of visitors read the panel on the nursery door building and then decided to enter or not based on both the type of species and the additional fee. We obtained an average of 112 persons per day per combination of the type of species and fee. The percentage of people entering into the nursery was significantly related to the type of species (table 2) and the fee (*Χ*^2^=53.49, *p*<0.001). As the fee increased, less people were willing to pay to enter into the nursery (table 1; figure 1*e*). Yet more of them were willing to pay to see the rare species (e.g. 3% of people agreed to pay the highest fee to see the rare species but none to see the common species).

### (f) Risk exposure to get a rare species

Although the seed display was set up so that stealing would be difficult (in full view, almost out of reach and behind a fence), 1185 seeds were stolen in 25 days. More rare seeds were stolen than common seeds (tables 1 and 2; figure 1*f*). The position of the jar did not affect the number of seeds stolen (*Χ*^2^=0.16, *p*=0.691).

## 4. Discussion

### (a) Validating the AAE theory: rare species are more valued

The AAE theory states that if people did value rare species, there would be no economic constraint to the exploitation of species at low density, as even high exploitation costs would be surpassed by high prices when the demand can be sustained, or even augmented, as rarity increases (Courchamp *et al*. 2006). The outcome of the AAE is an increased threat of extinction for the rare species, simply because they are irrationally valued more than the common ones. We aimed here to assess whether people did value rarity, an assumption that underpins the whole AAE theory. We demonstrate that when presented with a choice between a rare and common species, people were more interested by the rare species.

Courchamp *et al*. (2006) described the AAE with a mathematical model and a set of examples of possible activities linked to this effect. If these examples did not allow a distinction between correlation and causation, the experiments proposed here overcame these problems by comparing the value attributed to rare and common species. We also solved the cross-species comparison problem by performing most of the experiments without giving the name of the species that visitors were going to access. Because experiments were based on the two panels for which exactly the same (lack of) information was given, it is actually the difference between the value of the rare and the common that we studied (potential bias are similar for the two panels). The independence of our results from specific species confers higher relevance and generalization to our conclusion.

Although our experiments were carefully designed to avoid biases, some weaknesses could exist, e.g. the population sample that was concerned. Our experiments were performed in only one zoo situated in Paris; even if Paris represents a cosmopolitan city with people of mixed cultures, country-specific cultural roots could be biasing our results (Torgler & García-Valiñas 2007). Yet there is *a priori* no reason to believe that the visitors of this historical zoo (the oldest in France) would attract a sample of visitors differing from other zoos (experiments were conducted in the periods of high tourism). The World Association of Zoos and Aquariums claims that more than 600 million people visit zoos and aquarium worldwide each year (http://www.waza.org/network/index.php), i.e. nearly 10 per cent of the entire human population. Although these striking figures may need to be taken with caution, it is clear that zoo visitors represent a very large proportion of the inhabitants of the industrialized countries—those that are likely to constitute the demand in economic markets, which creates an AAE. Also, we did not record variables such as gender, age, profession or income level, which could have given valuable information on the socio-economic correlates of the tendency to attribute value to rarity. This is simply because the experiments required that the subjects were not aware that their reactions were being observed, which would have otherwise introduced a behavioural bias in the study.

### (b) How to measure the value of a species? Personal investment

Previous work has mainly focused on the perceived value of rarity in relation to the economic value, directly relating rarity with the price people actually pay (Courchamp *et al*. 2006) or relating species value with willingness to pay (see Martín-López *et al*. 2007). However, willingness to pay is currently a controversial measure for the value people allocate to species of conservation concern (Martín-López *et al*. 2007). Non-monetary criteria have been proposed such as the ones derived from social or psychological disciplines (Martín-López *et al*. 2007). Ojea & Loureiro (2007) stated that differences between individual environmental attitudes, perceptions of the environmental problems and prior informational levels can affect willingness to pay estimates, which may be more influenced by these ethical variables than by the respondent's other socio-economic characteristics.

In this paper, and for the first time, we propose a gradient of investment with different metrics to estimate how much the general public valued rare species by comparison with common ones. We assumed that the value was proportional to personal investment, and estimated such investment in sequential experiments: time spent in recreation and searching, physical effort, unpleasantness, economic investment, and risk of being caught while thieving. All measures were significantly increased when the species concerned was rare. The consistency in this trend between experiments lends greater support to their validity. Yet these measures are rather original and would benefit from some validation of their adequacy, which is not simple without venturing into psycho-sociological studies (see Martín-López *et al*. 2007; Ojea & Loureiro 2007).

In addition, it is also likely that people would claim a willingness to invest more than they actually would. For this reason, we did not limit our study to asking what visitors would be willing to do (which we actually did prior to this study, questionnaire results not shown). We instead put the visitors in situations where their actions, or lack of actions, would unambiguously indicate (and quantify) their willingness to spend time, effort and money, or take a risk, for a given species. In addition, the economic investment experiment relied on actual (not hypothetical) fees, as people entering into the nursery had already decided to pay (many of them had the money already prepared). The fees were actual, but not cashed, and could thus be more accurately described as a ‘readiness to pay’ than a ‘willingness to pay’. Because the visitors were not aware that their decisions were being monitored, we believe that these parameters were not biased and reflected the real relative value these visitors attributed to a given species.

### (c) Implications for the conservation of rare species and future research

The demonstration of the higher value given to rare species in comparison with common ones is important for the conservation of countless species. The vulnerability of some rare species has been well publicized in the cases of charismatic species, but many others are affected. In addition, many others could become rare in the near future and some could rapidly become rare simply because they become fashionable in one market or another.

The evidence that people value rarity can be exploited in two opposite ways. The first way is of real concern and has been under way for some time: a number of well-organized markets take advantage of the higher value consumers bestow on rarity to develop and sustain the legal or illegal trade of wild plant and animal species. These markets are so diverse as to include bird eggs, insects, orchid or seashell collections, luxury food and other goods, exotic pets or trophy hunting (Courchamp *et al*. 2006). They are so profitable that wildlife trade is now considered the second largest direct threat to species survival, after habitat destruction. TRAFFIC, the joint wildlife trade monitoring network of the World Wide Fund for Nature (WWF) and the World Conservation Union (IUCN), has calculated that wildlife products worth approximately US$160 billion were imported around the globe each year in the early 1990s (WWF 2006) and the wildlife market has only been increasing ever since. The legal trade alone involves hundreds of millions of wild plants and animals from tens of thousands of species. In addition to this, there is a large but unquantifiable illegal wildlife trade. According to the WWF, the illegal wildlife trade may now be the second largest area of organized crime after drugs (http://www.wwf.org.uk/wta/wildlifetradeappeal.asp?pc=VBQ010013). As the illegal wildlife trade is in part driven by a demand for rare species, which are protected, the sheer volume of the species threatened by this type of trade is outstanding, and rare species are likely to be a disproportionately large fraction of them.

Another matter of concern is that the value conferred to rare species is such that worldwide wildlife trade, whether legal or not, is becoming increasingly organized, in particular at detecting species of interest. As an example, newly discovered species are rapidly spotted from the scientific literature and their populations are subsequently immediately depleted to fuel diverse markets, such as exotic pets (Stuart *et al*. 2006). The perceived rarity of species is also reflected by their status according to the Convention on International Trade in Endangered Species of Wild Fauna and Flora (CITES), which regulates or bans their trade, and a recent study has shown that the proposal to change species to a more protected status, because their rarity had made them more vulnerable to exploitation, resulted in an immediate and important increase in their trade (Rivalan *et al*. 2007). Advertising rarity without restriction when working for nature conservation is not an inconsequential action, as this could trigger ill-placed desire for exploitation that would be detrimental for the species concerned. For example, several botanical gardens, when giving public information about rare plants, have the policy of not displaying maps so that the public cannot locate them. Similarly, the birdwatcher community has self-imposed rules in this regard, and information on the location of rare local birds is not authorized in some countries. This type of information filter would be quite important and effective in a great number of cases. Yet information on rarity can, on the contrary, be an ally in the struggle against biodiversity loss.

Indeed, the second way of exploiting the knowledge that people do value rarer species is its use for the conservation of these species. It is surely important to know that, all other things being equal, conservation programmes for rare species have the potential to be the target of public support only because of the value their rarity confers them. Thus, focusing on rare species when searching for public money could be an advantage to fund-raising or area protection in a number of cases. This is good news for conservation and should be exploited for the protection of not only the rare species but also the ecosystem they live in, making rarity an attribute for both umbrella and flagship species.

In any case, the origin and specifics of this irrational preference for rare species is a key to understand and tackle the resulting behaviours, be it fighting wildlife trade or encouraging wildlife conservation. This remains to be deciphered through socio-psychological studies, which are now strongly needed both to understand the origin and conditions of this preference, and the possibility to act either against it or with it.

## Figures and Tables

**Table 1:** Effort of the visitors (mean±s.e.) for the rare and common species as observed for each experiment. (Mean values relate to the number of visitors who have made or were ready to make efforts, when relevant.

experiment	rare	common
*observation*		
time of observation (s)	17.22±0.41	13.7±0.32
visitors who observed	608	608
*searching for the frog*		
time of searching (s)	11.83±0.54	6.05±0.35
visitors who searched	178	178
*searching for the gecko*		
time of searching (s)	12.28±0.72	6.57±0.49
visitors who searched	113	110
*physical effort*		
number of floors	1.53±0.06	1.47±0.08
visitors ready to climb	309	177
total number of visitors	444	412
*tolerance of unpleasantness*		
visitors sprinkled	177	62
total number of visitors	373	324
*economic investment*		
quota to pay	2.03±0.17	1.3±0.12
visitors ready to pay	81	28
total number of visitors	428	467
*risk exposure*		
number of seeds stolen per day	29.76±6.28	17.64±4.15
total number of seeds available per day	500	500

**Table 2:** Main statistics of each experiment (*F* or *Χ*^2^) and significance levels associated (*p*_1_), intercepts, parameter estimates, standard errors (s.e.) and significance levels (*p*_2_) of the rarity effect for each experiment. (Parameter estimates refer to the common species over the rare; parameter estimate for the rare=0. In the observation experiment, cases 1–4 are described in the legend of figure 1. The *n* refers to the number of visitors for each experiment, except for the risk exposure experiment, where it refers to the number of days.)

experiment	*n*	*F* or *Χ*^2^	*p*_1_	intercept	parameter estimates	s.e.	*p*_2_
observation							
case 1	230	27.68	<0.001	2.576	0.212	0.037	<0.001
case 2	286	119.17	<0.001	3.023	−0.558	0.036	<0.001
case 3	36	2.07	0.150	2.565	−0.149	0.103	0.146
case 4	56	7.92	0.005	2.974	−0.273	0.089	0.002
searching for the frog	355	156.05	<0.001	2.443	−0.621	0.059	<0.001
searching for the gecko	213	81.22	<0.001	2.510	−0.631	0.069	<0.001
physical effort	856	106.03	<0.001	0.466	−1.161	0.115	<0.001
tolerance of unpleasantness	1369	106.03	<0.001	0.089	−1.506	0.218	<0.001
economic investment	895	26.95	0.014	−3.742	−1.490	0.307	<0.001
risk exposure	25	11.19	<0.001	3.454	−0.519	0.070	<0.001
